# Reimagining falls prevention with insights from systems mapping on the use of millimetre-wave radar for remote health monitoring

**DOI:** 10.1038/s41598-025-14416-y

**Published:** 2025-08-07

**Authors:** Elif Dogu, José A. Paredes, Akram Alomainy, Janelle M. Jones, Khalid Z. Rajab

**Affiliations:** 1 School of Electronic Engineering and Computer Science, Queen Mary University of London, London, E1 4NS UK; 2 School of Arts, Humanities and Social Sciences, University of Roehampton, London, SW15 5PU UK; 3 Department of Biological and Experimental Psychology, School of Biological and Behavioural Sciences, Queen Mary University of London, London, E1 4NS UK

**Keywords:** Ambient sensing, mm-wave radar, Falls risk assessment, Health informatics, Digital health technology tool, Fuzzy cognitive maps, Electrical and electronic engineering, Preventive medicine, Risk factors, Geriatrics

## Abstract

Falls constitute a significant public health concern, demanding innovative solutions that transcend traditional methodologies. Current falls practice focuses on reactive post-fall assessment and management rather than proactive prevention and mitigation. We propose that millimetre-wave radar technology for real-time, continuous falls risk screening at home may address the limitations of current falls practice. To investigate the feasibility of this solution, we interviewed five experts in physiotherapy, falls prevention among older adults, and comprehensive geriatric assessment to identify the current state of play and potential for changes to falls practice. We applied a novel technique, systems mapping, to visually illustrate and analyse the interactions between components of current and proposed systems for addressing falls and constructed two conceptual maps: First, the current system was mapped by asking experts about the causal relationships between 15 system components. Second, to examine the feasibility of the proposed system, the components related to falls risk screening were replaced by radar-based home monitoring and experts were asked to re-evaluate the causal relationships between system components. Next, four scenarios (no fear of falling, no mobility limitation, maximising screening in the current system, maximising radar-based screening) were applied using the maps. Experts identified mobility deterioration and previous falls as key indicators of future falls, noting that increased screening in current practice could reduce risks but increase healthcare professionals’ workloads. Experts were positive about radar-based wireless home monitoring, believing it could reduce fall risks whilst reducing all fall-related costs. These findings suggest that, according to experts, millimetre-wave radar can be an effective solution in advancing falls prevention.

## Introduction

As the second leading cause of unintentional injury and deaths worldwide, falls are a major global health problem^1^. People aged 65 and older have the highest risk of falling, with 30% of people older than 65 and 50% of people older than 80 falling at least once a year^2^. A potential increase in the number of falls is of concern as the proportion of older adults at risk of falling will also increase. The number of people aged 65 years or older is projected to rise from 10% to 17% of the global population by 2050 (i.e., from 765 million to 1.6 billion people^3^). The human cost of falling includes distress, pain, injury, loss of confidence, loss of independence and mortality^2^. Falls are also associated with substantial financial costs. In the United Kingdom, the cost of falls is estimated at £2.3 billion pounds per year (equivalent to 2.9 billion USD per year)^2^, with unaddressed fall hazards in the home estimated to cost 435 million pounds (equivalent to 551 thousand USD)^4^. Moreover, the total annual social care cost of fragility fractures is estimated as 1.1 billion pounds (equivalent to 1.4 billion USD^4^). These figures highlight the growing financial burden of falls on both healthcare systems and society.

Despite the growing prevalence and financial burden of falls, ways to identify and proactively address falls have seen little innovation. Current falls practice encompasses three key steps to ensure a thorough evaluation and targeted intervention^2,5,6^. The initial falls risk screening classifies individuals as low, medium, or high risk using subjective assessments, questionnaires, and physical evaluations by healthcare professionals. For those individuals identified at risk, a comprehensive multifactorial risk assessment examines factors like gait, balance, frailty, comorbidities, medications, visual impairment, and environmental risks. Based on these findings, targeted interventions and management strategies are designed, including exercise programs, home modifications, and medication adjustments.

Although it is comprehensive, current falls practice has several limitations. First, there is a predominant focus on reactive post-fall assessment and management rather than proactive falls prevention and mitigation. The three-step-process initialises only after the first fall, which is problematic because these often lead to a subsequent fall^7^, making screening after the first fall too late. Proactive measures are essential to prevent the initial fall, thereby reducing the risk of severe injury, subsequent falls, and associated health complications. Second, falls risk screening in healthcare settings captures only a snapshot of the patient’s condition at a single point of time during the day, potentially overlooking other moments of vulnerability. Indeed, falls are frequently underreported ^8,9^ and weak associations between natural gait speed and in-laboratory gait speed have recently been reported^10^. Relying solely on reported falls or laboratory assessments may not capture the full spectrum of falls risk, hindering the effectiveness of interventions. Lastly, intervening only after a fall has happened misses the crucial opportunity for early prevention, often resulting in more severe health outcomes and increased healthcare costs. Addressing these limitations is essential to improve fall prevention strategies and enhance the safety and well-being of older adults.

Digital fall risk assessment protocols with wearable devices have been proposed to measure gait parameters^11–13^. However, wearables for continuous monitoring are challenging due to cost-related^14^, psychological^15^ and physical^16^ barriers to their adoption by older adults. Willingness to continue using wearable devices is influenced by several factors, such as reliability, self-perceived age, and usefulness^17^. Consequently, remote health monitoring systems that do not require user interaction, such as those using ambient sensors, offer a more promising approach for continuous monitoring in home settings^18^, as they eliminate the need for wearable devices while still providing valuable health data.

**Fig. 1:**
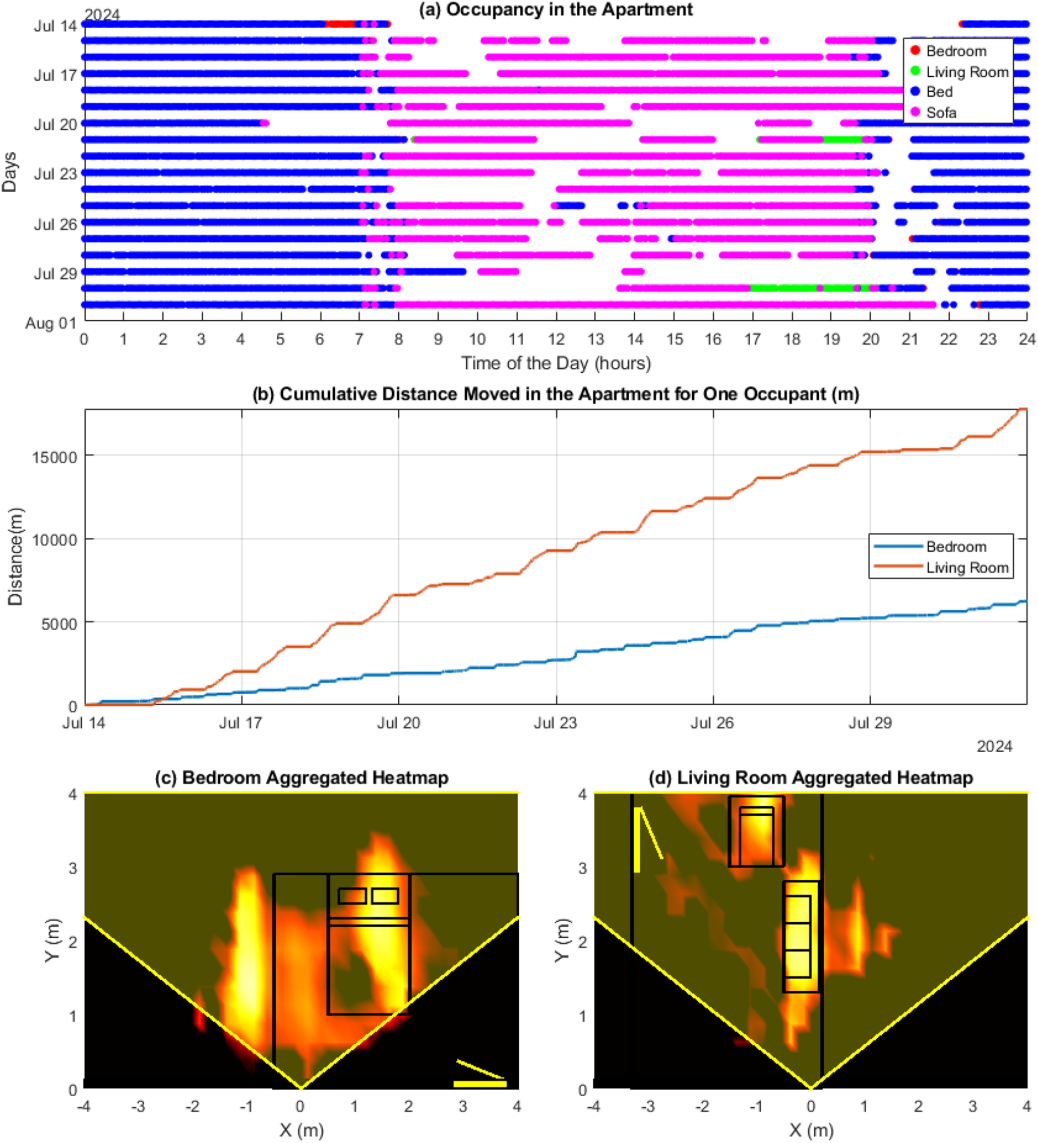
Activity analysis in an apartment. (**a**) shows occupancy in the Bedroom and Living Room over two weeks in July 2024. Red and green bars indicate time spent in each room, while blue and magenta markers show Bed and Sofa use, giving insight into daily activity patterns, such as sleep times and inactivity. (**b**) illustrates total distance travelled in both rooms, with more movement in the Living Room (red) compared to the Bedroom (blue). (**c,d**) Present heatmaps of movement, showing frequently occupied areas. The sensor was placed at position (0, 0) and was directed along the y-axis. Bedroom heatmap reveals that the occupant typically sleeps on the right side, useful for equipment placement and keeping that area obstacle-free. The apartment is on the ground floor, and outside the window of the bedroom there is a frequently used pathway, which is also captured on the heatmap ($X<0.5$ m). Similarly, in the Living Room (**e**), occupants are most often sat on two, or moving diagonally along the central walking area, between the seats and the entryway. We note that the sensors were placed flush to the wall, and the entirety of the room was not within the radar capture area – indicated by yellow lines. In future, the sensors should preferably be mounted at an angle to the wall to optimise radar coverage.

**Fig. 2:**
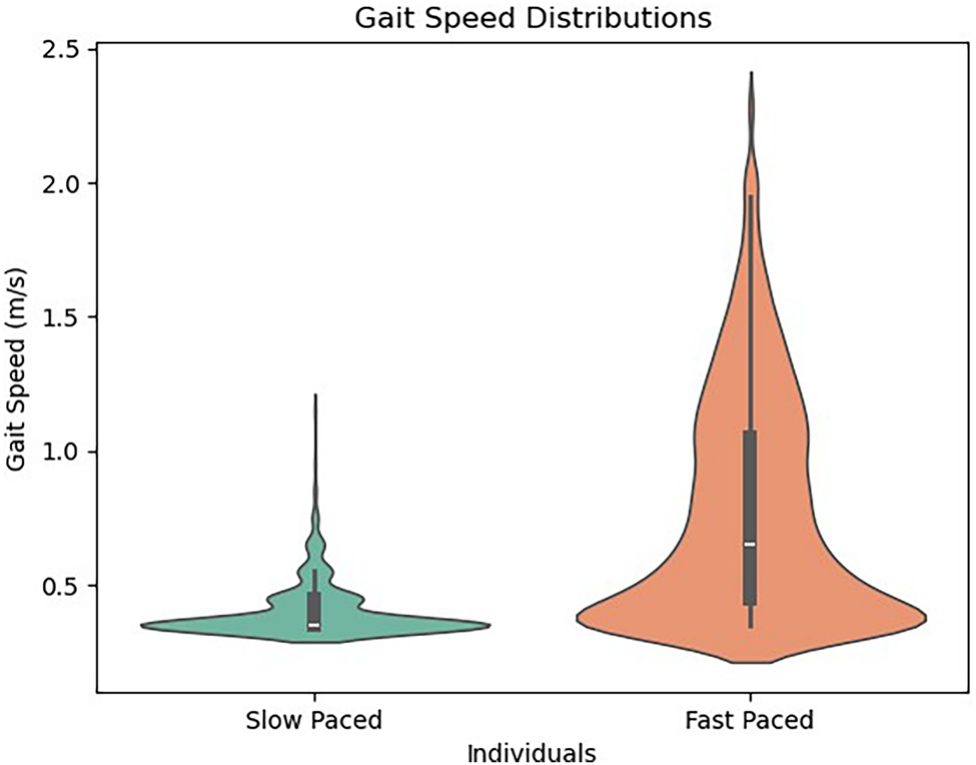
Comparison of gait speed between two individuals with slow and fast walking paces. For the slow paced individual, the median gait speed is 0.35 m/s, which is relatively low, suggesting slower and possibly less frequent movements. For the fast paced individual, the median gait speed is higher at 0.65 m/s, indicating more frequent or faster movements in comparison.

In this paper, we describe an ambient home sensing system using millimetre-wave radar technology for real-time and continuous falls risk screening. Our ultimate objective is to improve falls practice by implementing radar technology as a data-centric digital health technology tool^19^. We introduce a set of activity-related measures that are obtained contactlessly by millimetre-wave radar sensors at home. Millimetre-wave (mm-wave) radar technology is minimally intrusive, does not rely on wearable devices, and does not capture camera images, or videos. Once installed, it should not require any further action from the user. Frequency Modulated Continuous Wave (FMCW) radar is a type of radar system that can capture subtle movements with high precision. Our system comprises mm-wave FMCW radar sensors designed to emit high-frequency chirp-like signals within the GHz range. These radars have been used as effective tools combined with artificial intelligence (AI) algorithms for classifying daily-life activities such as walking, sitting, standing, and sleeping^20,21^. They have been shown to be effective as an automated falls risk screening tool^22^. Leveraging radar data, we extract target characteristics such as position, direction, and velocity. We infer several gait and mobility parameters as digital biomarkers^23^, which can separate fallers from non-fallers, such as gait speed, and activity levels^24^. Figure 1 illustrate representative samples of the parameters collected and the corresponding analyses performed to assess various aspects of mobility. Figure 2 depicts the differences between slow-paced and fast-paced walkers by comparing the relative distribution of their gait speeds. A detailed description of the proposed system, including an explanation of the measures, is provided in the Supplementary Information.

In this work, we begin by presenting a comprehensive exploration of current practice from a systems perspective, in which we see and understand systems as wholes, rather than the sum of its parts. This is used to develop a deeper understanding of underlying dynamics, and is suitable for improving a system with a transformative technology. We employed fuzzy cognitive maps (FCMs)^25^ as a knowledge extraction technique. FCMs are known for their ability to model complex problems^26^ and reveal causal relationships of systems in the medical domain^27^. It is a semi-quantitative method that involves comparing only the relative ranking of factor values^28^. Since it does not necessitate quantification or associated data, it allows for capturing expert knowledge without empirical evidence. Using FCMs, we interview experts to build a map of the current system, which is a causal model that represents the elements that make up the system and their relationships, showing how a change in one affects the other. Afterwards, by replacing the factors related to falls risk screening with radar-based home monitoring on the map, we discuss with experts its transformative potential in falls prevention.

To the best of our knowledge, although there is a large volume of published studies observing the risk factors and consequences of falls, there has been no previous attempt to reveal the causal relationships between them within a systems view. Therefore, the main contributions of this paper are as follows:

1. Presenting a systems map of the falls risk assessment and prevention system, demonstrating its key factors, underlying dynamics, and the quantification of well-established relationships with the help of domain experts—relationships that are often difficult to quantify through traditional data collection methods.
2. Proposing a millimetre-wave radar-based digital health technology tool for real-time and continuous falls risk screening at home and demonstrating its feasibility in the complex management of falls prevention.
3. Providing a foundation for further mathematical operations and scenario analyses, allowing researchers to explore system dynamics over the long term and assess the impact of different interventions.

This study aims to bridge the gap in falls prevention research by documenting expert knowledge with a systems approach, enhancing our understanding and management of falls risk, and paving the way for more proactive prevention strategies using transformative technology.

## Results

### Key concepts of falls prevention

We defined 15 key concepts of the current system as variables, following guidance on key concepts identification^28,29^ and proposed *radar-based home monitoring* for improvement (see Table 1 for a summary). The detailed definitions of key concepts are provided in Supplementary Information.

**Table 1:** Summary of the key concepts.

Key Concept	Definition
Risk of falls	The hazard linked to an individual’s susceptibility to falling.
Severity of previous falls	The level of severity of previous falls. A fall is severe if it has one of the five characteristics: (1) accompanying injury, (2) multiple falls in the last year, (3) known frailty, (4) inability to get up after the fall without help for at least an hour, and (5) accompanied by (suspected) transient loss of consciousness. Older adults who have fallen in the last 12 months had a greater history of falls than non-fallers ($71.9\%$ vs. $11.9\%$, $p<0.001$)^30^.
Fear of falling	Individual’s concern about falling. Evaluations of concern about falling are assessed using standardised measures such as the Falls Efficacy Scale International (FES-I^31^) or Short FES-I^32^ in community-dwelling older adults. A history of prior falls is associated with a twofold likelihood of experiencing fear of falling compared to individuals without such incidents^33^.
Adverse health conditions	All risks factors of falls related to a person’s health, other than mobility issues. They include problems with sensory functions (dizziness/ vestibular, vision, hearing), cognitive functions (cognition, delirium, behaviour), autonomic functions (orthostatic, hypotension), medications/polypharmacy, nutritional status^34^ (malnutrition, obesity, vitamin deficiencies, substance abuse, alcohol use), and disease history such as cardiovascular disorders, contributing diseases (diabetes mellitus, osteoarthritis, neurological disorders), atypical disease presentations of acute conditions (e.g., pneumonia), Parkinson’s disease, and depression^6^.
Activities of daily living (ADL)	The extent to which a person can successfully perform ADLs (e.g. ambulating, feeding, dressing, personal hygiene, continence, and toileting) and Instrumental ADLs (e.g. transportation, shopping, managing finances, meal preparation, home maintenance and managing medications)^35^.
Mobility limitations	The problems related to mobility such as balance disorders, gait impairment, muscle weakness, frailty, the need for walking aid and potential foot problems (excluding footwear)^6,30,36,37^.
Environmental risk	Factors contributing to falls in relation to footwear and the indoor home environment^38,39^.
Activity level	The extent to which an individual engages in physical activity within the scope of their daily life^40,41^.
Falls risk screening	The number of falls risk screening procedures and assessments of a person conducted by a healthcare professional. The use of TUG test and measuring gait speed are recommended by the guidelines^6,42^.
Fall reporting accuracy	An individual’s accuracy in reporting the time and severity of falls (decreases in case of underreporting^8,9^).
Opportunistic finding	The observation of a “fall in the last year” by chance, during another visit to a healthcare facility^5,6^.
Effectiveness of intervention	The degree to which implemented interventions successfully reduce the incidence, severity, and consequences of falls, promoting overall well-being and preserving functional independence.
Primary costs to NHS	The financial expenditures incurred for medical services, including hospital stays, physician fees, medications, and treatments, associated with addressing fall-related incidents among individuals within the healthcare system^2,4^.
Healthcare professionals’ workload	The extent of responsibilities, tasks, and time commitments required of medical practitioners^43^, including doctors and nurses, in managing and addressing the healthcare needs, assessments, and interventions related to falls and associated consequences.
Societal costs	The broader economic and social impacts resulting from falls among older adults including social care^4^.
Radar-based home monitoring	The application of millimetre-wave radar technology within home environments to measure digital biomarkers systematically and continuously such as gait, mobility, activities, and behavioural patterns

We consulted the guidelines that healthcare professionals follow in their practice to define the key concepts of the system. These included National Institute for Health and Care Excellence (NICE) guidelines and quality standards^2,44^, World Guidelines for Falls of British Geriatrics Society^6^, American Geriatrics Society Response to World Falls Guidelines^42^, and Stopping Elderly Accidents, Deaths & Injuries (STEADI) resources of Centers for Disease Control and Prevention (CDC)^5,37^.

All experts agreed that the list of key concepts was comprehensive enough to represent the fall prevention system. They asked to ensure that three factors were included:

1. Polypharmacy (included in *Adverse health conditions*)
2. Continence (included in *Activities of daily living*)
3. Patient behaviour & engagement (included in *Effectiveness of intervention*)

### Causal relationships

**Fig. 3:**
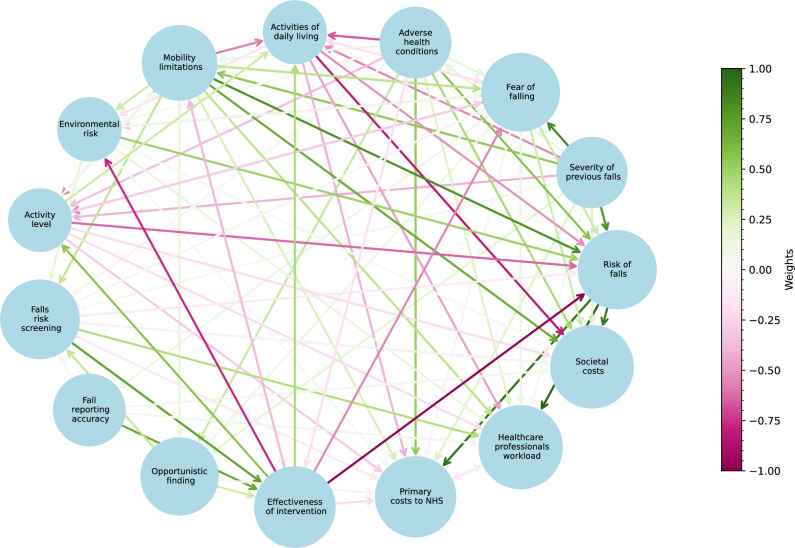
The dynamics of the current falls risk assessment and prevention system.

**Fig. 4:**
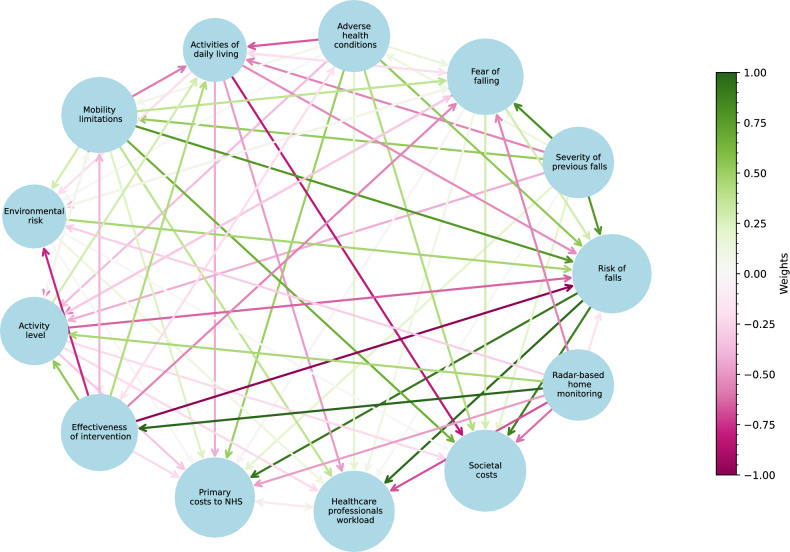
The dynamics of the proposed system replacing falls risk screening variables with radar-based home monitoring.

The maps of the current fall prevention practice and the proposed system with millimetre-wave radar technology are depicted in Figs. 3 and 4. Their adjacency matrices are provided in the Supplementary Information. During the interviews, while switching from the first map to the second, none of the experts changed their previous links on the map. Therefore, the two matrices have the same values for the same pairs of concepts, but the second matrix has an additional node *Radar-based home monitoring* that replaces three screening variables. The total number of connections were 90 (59 positive, 31 negative) and 72 (41 positive, 31 negative), respectively, for the first and second maps.

For the first map of the current system (Fig. 3), the strongest positive causal relationships were from *Risk of falls* to *Healthcare professionals’ workload*, *Primary costs to NHS* and *Societal costs* with weights 0.78, 0.74 and 0.71. All experts agreed that falls are a financial burden and increase their workload. The subsequent strong positive links were from *Severity of previous falls* to *Fear of falling* and to *Risk of falls* with weights 0.67 and 0.63 and from *Mobility limitations* to *Risk of falls* with 0.64. The experts stated that a deterioration in mobility and a previous fall in the last year were two main indicators of a future fall. The strongest negative connection was from *Effectiveness of intervention* to *Risk of falls* with weight -0.66, which was expected because interventions have been specifically designed by healthcare professionals to fight against falls. The second was from *Activities of daily living* to *Societal costs* with weight -0.54. Experts declared that ADLs were key determinants of older adults’ care needs.

For the second map of the proposed system (Fig. 4), the strongest positive connection was from *Radar-based home monitoring* to *Effectiveness of intervention* with weight 0.8. All experts agreed on the benefits that radar monitoring could offer them in designing targeted interventions and ensuring their continuous improvement. Moreover, *Radar-based home monitoring* had another positive link to *Activity level* and negative links to *Risk of falls*, *Fear of falling*, *Environmental risk* and to all three costs. A direct negative link from *Radar-based home monitoring* to *Risk of falls* was not expected, as assessing or measuring the probability of an event does not change its likelihood. However, a weak negative link was observed because one expert considered an indirect mechanism not explicitly represented in the map, suggesting that “the fact of being monitored could reduce the risk by increasing awareness,” potentially influencing behaviour.

### Fuzzy cognitive map measures

Table 2 shows indegree, outdegree, centrality, and final value measures (Please see Data Analysis in Methods) for both maps. *Risk of falls* had the highest centrality aligned with the objective of the map. Its indegree was greater than its outdegree which indicated that it can be sensitive to changes in the system. *Mobility limitations* and *Effectiveness of intervention* had high centrality as well, however, their outdegrees were dominant. Additionally, *Severity of previous falls* was the only node with zero indegree but with a high outdegree, which implied that these three factors could be sources of change in the system. The screening variables had the lowest centrality values, resulting in only minor changes in indegree, outdegree and centrality values in the second map, where they were replaced by radar-based home monitoring.

**Table 2:** FCM results of the current and proposed systems.

	Current System FCM Results				Proposed System FCM Results			
	Indegree	Outdegree	Centrality	Final value	Indegree	Outdegree	Centrality	Final value
Risk of falls	3.82	2.38	6.2	0.8166	3.86	2.38	6.24	0.8160
Severity of previous falls	0	3.29	3.29	0.6590	0	3.01	3.01	0.6590
Fear of falling	2.07	1.76	3.83	0.8062	2.37	1.52	3.89	0.7580
Adverse health conditions	0.24	2.92	3.16	0.6694	0.24	2.54	2.78	0.6716
Activities of daily living	1.92	1.54	3.46	0.5301	1.92	1.54	3.46	0.5265
Mobility limitations	0.99	3.65	4.64	0.7433	0.99	3.15	4.14	0.7489
Environmental risk	1.12	0.99	2.11	0.5372	1.26	0.81	2.07	0.5262
Activity level	1.58	1.29	2.87	0.5263	1.98	1.29	3.27	0.6025
Falls risk screening	1.05	1.04	2.09	0.8278	-	-	-	-
Fall reporting accuracy	0.12	0.84	0.96	0.6863	-	-	-	-
Opportunistic finding	0.8	0.60	1.4	0.7956	-	-	-	-
Effectiveness of intervention	1.53	2.74	4.27	0.8653	0.92	2.74	3.66	0.7708
Primary costs to NHS	2.3	0.16	2.46	0.8619	2.54	0.16	2.7	0.8347
Healthcare professionals workload	2.72	0.02	2.74	0.9253	2.69	0.02	2.71	0.8557
Societal costs	2.96	0.00	2.96	0.9090	3.22	0.00	3.22	0.8712
Radar-based home monitoring	-	-	-	-	0	2.83	2.83	0.6590

Considering the falls prevention system with the risk of falls at its centre, it was expected that the outdegrees of the risk factors for falls would be higher than their indegrees and vice versa for the factors that are outcomes of falls. Unsurprisingly, all three costs had higher indegrees. *Severity of previous falls*, *Adverse health conditions*, and *Mobility limitations* had higher outdegrees, according to experts, these were the main risk factors in the system. *Fear of falling*, *Activities of daily living* and *Environmental risk* had a balance, which implied that they could be both a cause or a consequence of falls under different circumstances.

**Fig. 5:**
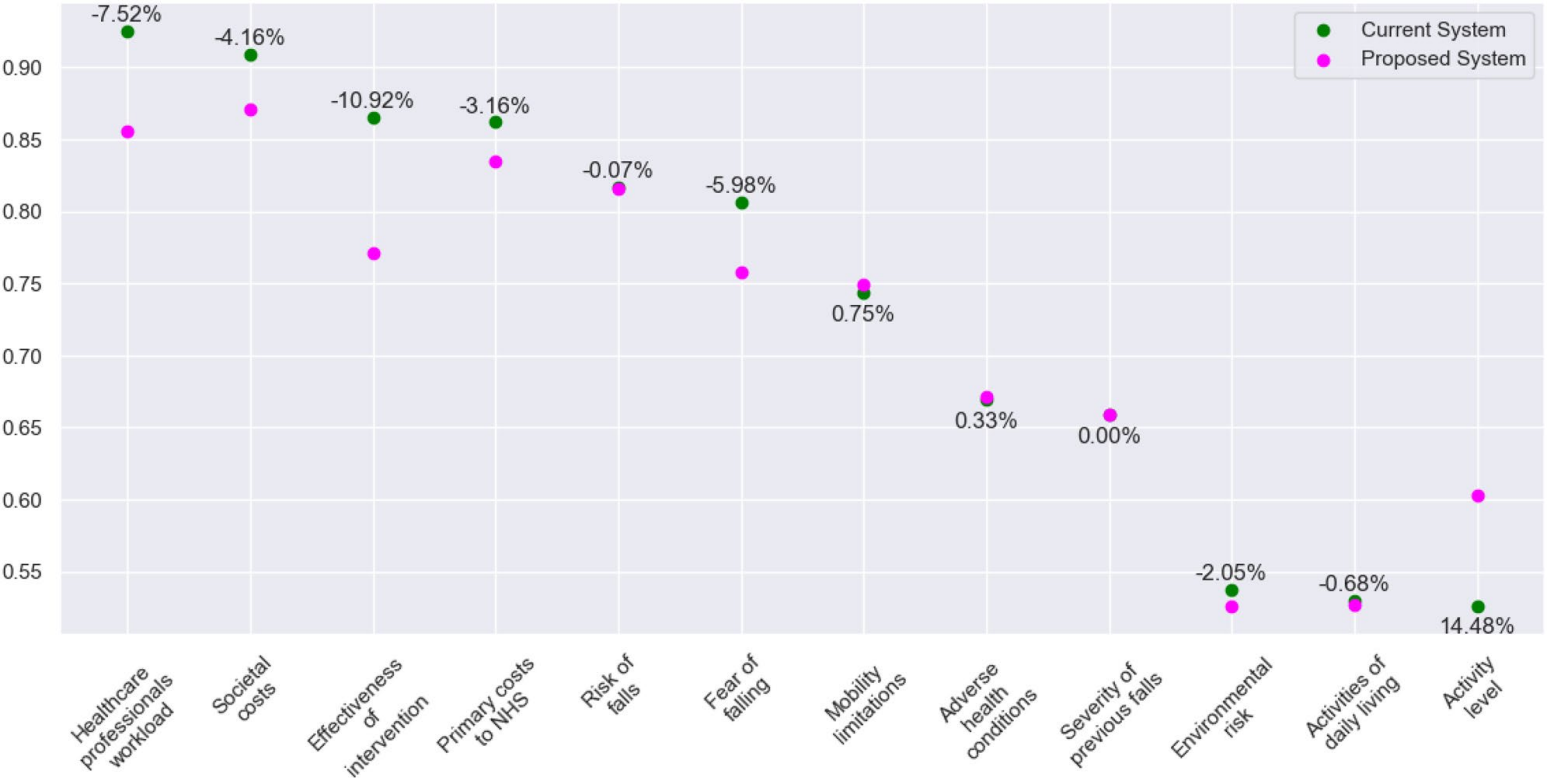
Final values of the concepts for current and proposed systems, and the percentage of change.

Figure 5 illustrates the differences in the final values of two maps, with the concepts arranged in descending order according to the values of the first map of the current system. The three cost factors and *Effectiveness of intervention* were identified as the most influential concepts in the system over the long term. The least influential factors were *Environmental risks* and two activity factors for both maps. The final value of *Risk of falls* exhibited a slight decrease (0.07%) in the proposed system. Meanwhile, the values of *Healthcare professionals’ workload*, *Societal costs* and *Primary costs to NHS* decreased by 7.52%, 4.16% and 3.16%, respectively. This indicates that the experts were of the opinion that the use of radar-based home monitoring would result in a reduction in the risk of falls over the long term. However, they were more confident that it would result in a reduction in costs and their workload. The largest changes between the two systems were a 10.92% decrease in *Effectiveness of intervention* and a 14.48% increase in *Activity level*. From a mathematical perspective, the second map eliminates three screening factors with positive links to *Effectiveness of intervention* and no link to *Activity level*. Considering that *Risk of falls* still tends to decrease, the results suggest that while *Effectiveness of intervention* will lose importance in the new system, *Activity level* will replace it. In the current system, there is no objective information regarding a person’s preferred level of activity at home, therefore, this factor is arguably of limited importance. In the new system with radar-based continuous home monitoring, where the healthcare professional is aware of a person’s lifestyle and level of activity, it is likely to become a much more important factor in terms of falls prevention. The final value of *Fear of falling* decreased 6.98% in the new system because all experts concurred that a radar-based monitoring system with fall detection would reduce a person’s fear of falling. *Environmental risk* had a decrease as well; three experts stated that knowledge of the precise time and location of the fall at home could be effective in preventing future falls.

The final values of both maps were all greater than 0.5 with no value significantly lower than the others. This indicated that the key concepts of the system have been selected effectively and there is no necessity to eliminate any of them. In these cases, the modeller must eliminate the concept with significantly less final value and repeat the analysis.

### Scenario analysis

**Fig. 6:**
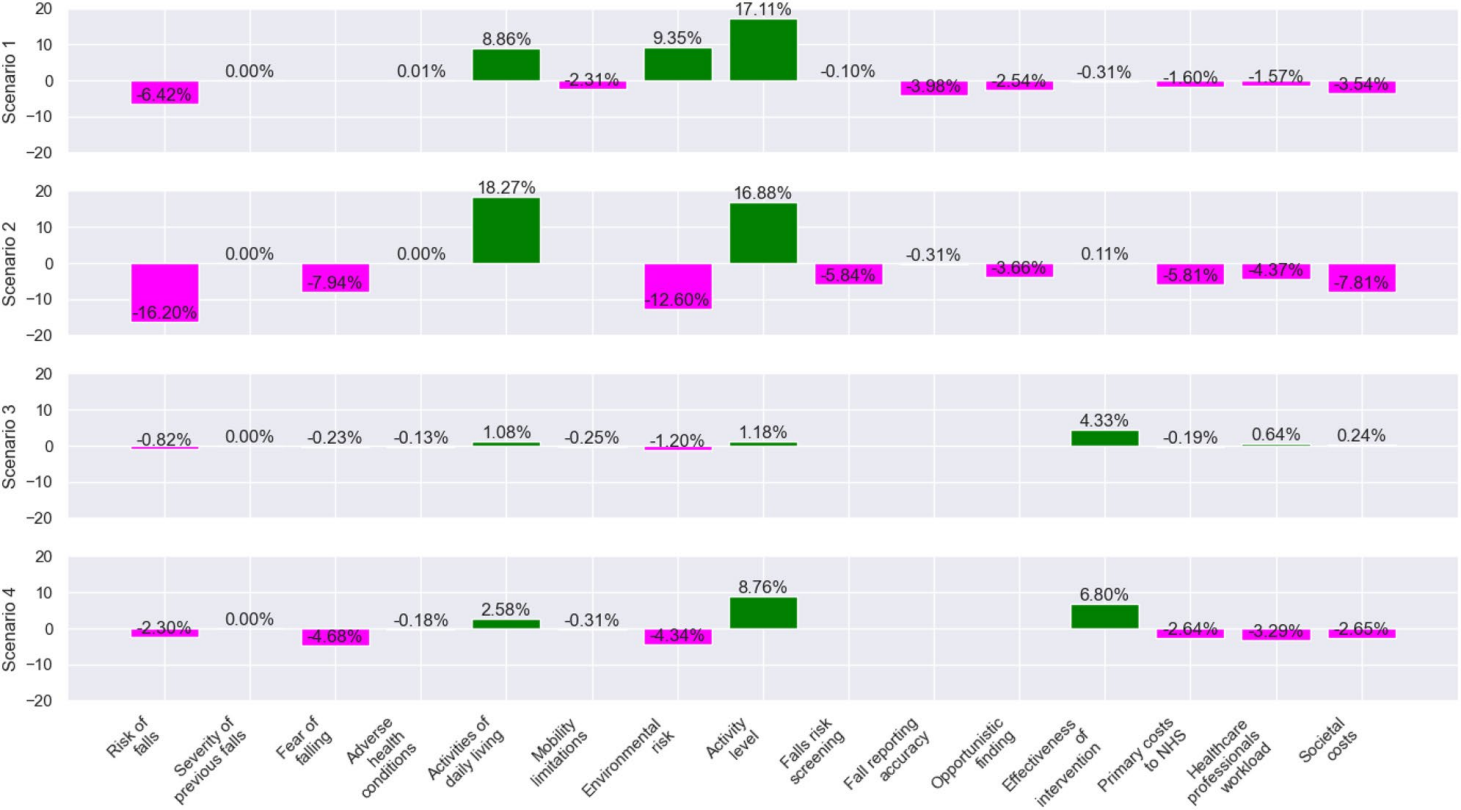
Changes in other system variables in different scenarios (Scenario 1: No fear of falling, Scenario 2: No mobility limitations, Scenario 3: Maximising fall risk screening in the current system, Scenario 4: Maximising radar-based fall risk screening.

FCM allows modellers to run what-if scenarios to observe how the system might react to different conditions. By setting the values of selected nodes to specific numbers between [0,1], modellers can simulate potential outcomes and consider their knock-on effects. This predictive capability can aid decision making in complex systems with non-linear interactions between variables. Four scenarios are selected to discuss the potential of continuous radar-based home monitoring within the context of falls prevention (see Fig. 6)

**Scenario 1: No fear of falling in the current system** The value of the *Fear of falling* node is set to zero in order to observe how the other system variables change if a person has no fear of falling. Having no fear of falling means that the individual reported “Not concerned” for all listed activities in the standardised measure of FES-I or Short FES-I, indicating an absence of self-reported concern about falling in daily life. Moderate reductions in risk of falls and societal costs, and large increases in both activity variables and environmental risk are observed. In the absence of fear of falling, people tend to be more active and more independent in their daily life, which means they would need less care. Their mobility limitations would decrease as well, since being active would improve their strength and balance, which would cause a reduction in two other cost variables. On the downside, it is possible that it could escalate the environmental risk and corrupt the reporting accuracy. Being more confident might result in a reluctance to take precautions in the home environment and to visit healthcare facilities.

**Scenario 2: No mobility limitations in the current system** This scenario assumes that the person has no mobility limitations. In this case, a notable reduction in the risk of falls and a large increase in both activity variables are observed. Since the fear of falling is decreased as well, the results of scenarios 1 and 2 have similarities. However, unlike scenario 1, the number of falls risk screenings and opportunistic findings was considerably reduced. In the current practice, a falls risk screening is conducted predominantly after the first fall or in the event of mobility-related issues. Furthermore, a larger decrease in all cost variables is observed. Therefore, the second scenario suggests that according to experts, in a situation in which costs due to falls are to be reduced, actions that focus on mobility limitations rather than fear of falling would be more efficient. Lastly, a steep decrease is observed in the environmental risk. One expert explained this effect during the interview: *“My feeling is that actually the vast majority of environmental risk is mediated through mobility limitations. Environmental risk factors are only an issue if you have limited physical reserves to deal with them. Otherwise, all of us would be falling over all the time.”*

**Scenario 3: Maximising screening in the current system** In this scenario, all three screening variables are set to the maximum value of 1 to observe the consequences of a situation in the current system in which all fall events are accurately detected or reported and assessed by a relevant healthcare professional, rather than being underreported. Scenario 3 assumes the maximum values for three screening-related factors: *Falls risk screening*, *Fall reporting accuracy*, and *Opportunistic finding*. This represents the highest level of fall risk information obtainable through traditional clinical methods, where individuals undergo frequent screenings, accurately report all past falls, and all previous falls are identified through opportunistic findings. There is a moderate change in the effectiveness of intervention. It entails an increase in the workload of healthcare professionals to the same extent as the reduction in the risk of falls. This scenario suggests that in the current system the risk of falls could be slightly reduced by improving screening, but at the cost of increasing healthcare professionals’ workloads as well as societal costs.

**Scenario 4: Maximising radar-based screening** In the second map of the proposed system, setting radar-based home monitoring to the maximum value of 1 yields different outcomes compared to Scenario 3. Notable positive changes are observed in the activity variables and effectiveness of intervention. Millimetre-wave radar provides detailed information on indoor activity, and on inactive periods during the day. Such feedback serves to raise the individual’s awareness of their own behaviour, which may then lead to a positive change in their daily life in terms of activity. For instance, a person who learns that they remain seated on the sofa for three hours each day, while watching the same television programme at the same time, may begin to rise from their seat every hour. From the intervention side, the proposed system measures gait speed and other mobility parameters continuously and summarises this information for healthcare professionals. The experts stated that replacing the intermittent information they receive at a single point in time (only when they see the patient) with continuous data would enhance the effectiveness of intervention in the long term, as it would allow for more targeted and customised interventions. It is worth highlighting that the maximum level of home monitoring not only reduces the risk of falls, but also reduces all three costs of falls. This scenario suggests that, according to experts, the use of radar-based home monitoring could overcome the cost-risk trade-off, which is observed in Scenario 3.

## Discussion

We analysed and reported the opinions of falls experts on the underlying dynamics of the falls prevention system and the potential of radar-based home monitoring to improve this system. We extracted the domain knowledge from a systems perspective and documented it in a way that would allow further analysis of the long-term consequences of possible changes. The map of the current system revealed the reinforcing causal relationships that could help explain many aspects of this complex system such as the substantial increase in fall-related costs in recent years, the two-way effect of fear of falling, and non-linear effects of activity levels. The great benefit of this big picture view of falls prevention lies in its ability to help researchers and healthcare professionals develop new systems to fight against falls. The conceptual map of the proposed system, where the fall risk screening variables were replaced by *radar-based home monitoring*, revealed the potential of this technology for reducing the risk of falls and all fall-related costs simultaneously. Experts were confident that the proposed system would enhance their daily practice and be effective in preventing falls.

While the scenarios are helpful in simulating the long-term consequences of changes, it is actually the first map itself that reveals how complex the system of falls prevention is and how difficult it is to deal with its intrinsic dynamics. The non-linear relationship between the activity level and risk of falling was quite challenging for the experts to describe. One expert stated: *“One of the really important things about physical activity is that it changes your exposure to falling. We know that being active keeps you stronger and keeps you fitter and, theoretically reduces your risk of falling, but being active also increases your exposure to falling. What we are trying to do is to allow people to be as active as possible, having as few falls as possible”*. Since the radar-based home monitoring is designed for older adults who live alone, it serves the purpose of keeping them active by providing detailed and continuous information about activity levels within the home that they have not had before. This is why the presence of the *Activity level* concept is stronger in the proposed system map.

Another challenging aspect was the feedback loop between falls and the fear of falling^33,36^. On the system map, *Fear of falling* is influenced by *Mobility limitations* and *Activity level*, and reciprocates by influencing them. In experts’ opinions, mobility and activity levels of an individual can be the underlying reasons for this complexity because connections in both directions create feedback loops in the system. By addressing all the loop components simultaneously, radar-based home monitoring has the potential to improve the system. The results of scenario 4 indicate that the proposed system can increase activity levels while decreasing mobility limitations and fear of falling.

In addition to complexity, the current system suffers from missing data because falls are underreported^8,9^. Millimetre-wave FMCW radar technology can detect both sudden and soft falls with 90%-100% accuracy^45^. It can capture fine-grained motion and differentiate between various types of falls and other activities^20,21^.Moreover, it can provide detailed information on in-home gait speed distributions and variability, which have shown to be effective as digital biomarkers in predicting falls^46^.

Financial modelling of the cost of falls was another predicament, with experts suggesting that continuous home monitoring over longer periods could be beneficial. One expert explained: *“There’s not so much evidence for modelling the longer-term effects of having a fall. For example, they might have a fall and need an immediate medical treatment. But also, they might have a fall and just not feel confident, so they walk a bit less and they get a little bit frailer. In five years time, the consequence of having a fall is actually they’re frail, quite dependent and their functions are limited, which they might not have been, in theory, if they hadn’t had that fall. The impact that falls will have on longer term health declines, which is perhaps not captured in the immediate treatment cost of falls.”* Radar-based home monitoring can provide invaluable information in understanding the progressive impact of falls by reporting long-term mobility parameters such as changes in gait speed, gait patterns and overall activity levels. This information can help in modelling the wider economic and health impact of falls, supporting better resource allocation and planning in healthcare systems.

At an individual level, continuous self-reporting of mobility information can raise awareness and encourage proactive management of personal health risks. Recently, it has been reported that improving the accuracy of older adults’ perceptions of their own risk of falls and highlighting the fact that many falls are preventable are two key messages that can help motivate older adults to take action to prevent falls^47^.

This study carries inherent limitations of fuzzy cognitive map modelling. The two maps of the current and proposed systems were constructed based on expert opinion, which may introduce subjective bias into the model. In order to include all stakeholder perspectives, we recruited experts from different backgrounds, with different roles and from different settings. Our aim was to elucidate the information that cannot be inferred from data, but can only acquired by field experts over many years.

Another limitation was scalability. FCMs become harder to manage as the number of factors increases. We created groups of factors to have an easy-to-interpret and concise map; however, this could lead to some of the lower-level dynamics being overlooked. For example, the concept *Adverse health conditions* included many conditions such as dizziness, hypotension, polypharmacy, and malnutrition. Although we were aware that these might have different effects (arrows with different weights) on the risk of falling, we chose to present them as one factor, due to time limitations we had with experts and to reduce complexity. These individual effects should be explored in more detail to further refine the model.

This study forms the basis for the development of a remote health monitoring technology that aims to transform the falls prevention practice. By engaging with stakeholders through Patient and Public Involvement and Engagement (PPIE) activities, we ensured that the design of this technology is aligned with the real-world needs of both healthcare providers and patients. We introduced a novel approach to falls prevention by identifying the causal relationships between falls risk factors, informed by expert insights, and examined how millimetre-wave radar technology can be integrated into the current system. Future studies can focus on the actual deployment of this system and the assessment of its feasibility, as well as creating additional maps with different stakeholder groups, such as older adult patients, carers, and staff members from care facilities, to better understand their perspectives and priorities. Further refinement is necessary to ensure that radar-based home monitoring remains responsive to evolving needs and gains widespread acceptability.

## Methods

### Patient and public involvement and engagement

We conducted patient and public involvement and engagement (PPIE) activities to ensure our study addresses the requirements of healthcare professionals whilst remaining sensitive to the needs of patients and maintaining a balance in data generation. To do this, digital biomarkers, recorded through mm-wave radar, were selected to ensure that the information is both meaningful enough to support healthcare professionals in decision-making, and concise enough to not add to their workload.

Two PPIE activities shaped our study. The first engaged with older adult residents at a retirement village, residential staff, and NHS clinicians to identify the key issues that are being faced in monitoring older adults’ health and wellbeing, and their thoughts and opinions on different ambient monitoring technologies. Feedback indicated that residents preferred non-invasive sensors over wearables and felt additional monitoring would enhance their security. Staff members expressed concerns about the extra information such monitoring might entail.

The second PPIE activity included consultations with five falls prevention experts to gain insights into the practical utility of real-time and continuous monitoring. Experts highlighted several benefits, such as passive monitoring of daily activities, comprehensive gait analysis, prediction of risk periods, accurate fall diaries, and improved intervention efficacy. They unanimously recognised the potential of this technology to enhance their practice and patient outcomes by enabling a proactive approach to fall prevention.

These insights from PPIE activities guided the design and focus of our proposed home monitoring system. As a result, it is built using only contactless technologies with a customisable dashboard that summarises the information, allowing users to adjust the level of detail.

### Study design

This study employed fuzzy cognitive maps (FCMs)^25^ as a knowledge extraction technique to construct a conceptual model of the falls risk assessment and prevention system for older adults. The process of building FCMs consists of three steps^28^: (1) defining the factors/key concepts (nodes of the map); (2) constructing the map with causal relationships (arrows/edges of the map); and (3) conducting analysis and interpreting outcomes.

### Participants

The inclusion criteria for the participants required that they work as a healthcare professional and to have at least 10 years of experience with falls. Five experts with an average of 19.2 years of experience in falls were recruited, ensuring a high level of domain expertise. They all had both operational and managerial roles. They had experience in a variety of healthcare settings including primary care, community, nursing home, residential care, acute hospital, acute elderly care, medical ward, outpatient clinic and private providers. The number of experts was chosen based on previous FCM applications in medical systems^48–50^, which require specialised knowledge, and was determined by considering both their average years of experience and the need to avoid individual bias. All participants provided written informed consent by reading and signing a consent form that was approved by the institutional review board. This study was carried out in accordance with the guidelines proposed in the Declaration of Helsinki, and the study protocol was approved by the Queen Mary Ethics of Research Committee with reference number QME24.0295.

### Data collection - recruitment of participants

Our project partner — Health Innovation Network (HIN) South London — supported us in linking with healthcare professionals. When experts expressed their interest in participating, an introductory email was sent explaining the objectives and methodology of the study. Preliminary information was sent, which included Table 1 of key concepts, along with the questions that we would ask them during the one-to-one interviews (See Table 3).

**Table 3:** Questions of the semi-structured one-to-one interviews.

General 1	What is your job title and current work setting?
General 2	What are your areas of expertise?
General 3	How long have you been working in this field?
General 4	What settings have you worked in?
Fall Prevention 1	What do you think of this summary of key concepts (e.g., good/comprehensive; bad/incomplete)?
Fall Prevention 2	Are there any other significant factors that affect/are affected by falls risk that can be added to the list?
Fall Prevention 3a	For each two concepts, does a change in Concept 1 create a change in Concept 2? (Yes or No)
Fall Prevention 3b	What is the nature of this change? Is it positive or negative? Is there an increase or decrease? Something else?
Fall Prevention 3c	How certain are you about this change? / What is the strength of this relationship?

### Semi-structured one-to-one interviews

There are three main approaches to creating FCMs^28^: (1) researchers can construct them independently based on their chosen evidence, (2) build them collaboratively with experts through individual interviews that are then merged, or (3) create a single map in a workshop setting with multiple participants. In this study, the first author served as the modeller and conducted one-to-one semi-structured interviews with experts.

One-hour interviews were structured into three stages:

- Stage 1: The modeller explained the key concepts and their definitions to clarify the details and presented the proposed radar-based continuous home monitoring system.
- Stage 2: The modeller asked the above questions for the first 15 key concepts to build the map of the current system.
- Stage 3: The modeller replaced the variables *Fall risk screening*, *Fall reporting accuracy* and *Opportunistic finding* with *Radar-based continuous home monitoring* to build the map of the proposed system and asked the same questions for this new variable. During the conversations, the experts also shared their views on how they thought radar technology could improve the current system.

These interviews were facilitated by the web-based ‘mentalmodeler’ tool (https://www.mentalmodeler.com/)^51^. Both the mapping and matrix views were used to prevent confusion. While asking for the direction of causal relationship (Question 3b), we stressed the meanings of the words ‘positive’ and ‘negative’. The distinction between positive and negative connections in this methodology is mathematical (co-movement vs. inverse movement), rather than normative as good vs. bad influences. Regarding the strength of the relationships, the most important information is their relative strength compared to each other to gain insight into the system. For practicality purposes, we adjusted the general rules followed by^52^ for our application in the falls domain:

1. The maximum value of a causal relationship was set at 0.9, considering that there is no evidence in the literature for a complete link (1) between any of these key concepts.
2. The minimum value was set at 0.1. The relationships with a strength less than 0.1 were assumed 0.
3. Between the weakest (0.1) and the strongest (0.9) relationships, the participants were asked to choose on a continuous scale. No other linguistic variables were used. This provided them the opportunity to make mathematical comparisons between the previous links and refine their decisions more sensitively.

### Data analysis

The data collection phase from five experts provided two individual maps for each expert and their square adjacency matrices. First and second maps had 15 and 13 nodes, respectively. The mean aggregation operator was used for the mathematical aggregation of these matrices^53^. Since the experts had similar levels of expertise, no weights were assigned to their matrices. As a result, two aggregated adjacency matrices of average edge weights were obtained to represent the two maps of current and proposed systems.

Indegree, outdegree and centrality measures are employed to observe the relative causal presence of the nodes within the map. Indegree and outdegree are the sum of absolute weights of the links entering and leaving a node, respectively. While a high indegree indicates that the concept is heavily caused/influenced/driven by other factors and hence sensitive to changes in the system, a high outdegree suggests that the concept is a driving force and can be a source of change within the system. Centrality is the sum of the absolute weights of all the connections of a node, showing how central a node is within the system and how critical it is in the functioning of the system.

In addition to these, the FCM method uses an iterative process to reach the final values of each factor within the system. The final values are typically between 0 and 1 and can be interpreted as indicative of the degree to which the factor is present or caused in the system in the long-term.

The iteration process starts with the unit vector of all concepts $\textbf{V}$, and the values or concepts are updated at each iteration *i* using the Equation (1), which is the vector representation of the equation proposed by^54^:

1 $$\begin{aligned} {V}_i=\textrm{f}\,\bigl ({V}_{i-1}{W}_{\!a}+{V}_{i-1} \bigr ) \end{aligned}$$

where $\textbf{V}^i$ is the state vector of all concept values at iteration $\textbf{i}$, $\textbf{W}_{\!a}$ is the aggregated weight matrix of causal relationships, and $\textrm{f}()$ is the threshold function. The sigmoid function is utilised as the threshold (squashing) function, which bounds the factor values between 0 and 1. This function is chosen for its stability and robustness to noise, compared to the other threshold functions such as hyperbolic tangent, step and linear^55^. FCMappers software is used for the calculations^56^.

## Supplementary Information

Supplementary Information.

## Data Availability

The data used in this manuscript is provided in the Supplementary Information.
