# Supplementary material for: Reimagining falls prevention with insights from systems mapping on the use of millimetre-wave radar for remote health monitoring
Source: Sci Rep. 2025 Aug 7;15:28842. doi: 10.1038/s41598-025-14416-y (PMC12328740; doi:10.1038/s41598-025-14416-y)
Supplement: Supplementary file 1 — Supplementary Information. [file 41598_2025_14416_MOESM1_ESM.pdf]

Reimagining Falls Prevention: Insights from Systems Mapping on the Use of Millimetre-Wave Radar for Remote Health Monitoring

Supplementary Information

1. Millimetre-Wave Radar System for Real-time and Continuous Falls Risk Screening at Home

We followed clinical guidelines for parameter selection of our system. The British Geriatrics Society suggests using either gait speed or a Timed-Up-and-Go (TUG) test for falls risk screening[1]. The American Geriatrics Society (AGS) recommends continued use of the TUG test rather than gait speed[2]. Both guidelines suggest increasing activity levels as interventions. The parameters of "gait speed" and "activity level" are first included in our monitoring system. The parameter "room occupancy" is included for the calculation of these parameters and to collect information on behavioural patterns.

The TUG test involves a simple procedure, which is a reason for its popularity. A patient is requested to stand up from a chair, walk three meters forward, turn around, walk back to the chair, and then sit down. Digital health technology tools can collect data in two ways[3]: (1) sensor data is captured while the person performs specific tasks ("active tests"), or (2) sensors monitor human behaviour passively ("passive monitoring"). Our system offers passive monitoring. To model the TUG test using normal daily activities, two further parameters are defined: "get up and go time" and "return to seated position time".

"Get up and go time" is delineated as the duration from the moment an individual initiates standing up until they traverse a specified distance within the room. The extent of this distance is contingent upon the room's dimensions. "Return to seated position time" denotes the inverse process, wherein the individual enters the predefined area and sits down. These two parameters encompass most of the TUG test, except for executing a 180-degree turn between them.

The last four parameters are included to ensure precise reporting of falls: "fall detected", "timestamp", "fall location", and "duration on the floor". Whenever a fall is detected, it is documented along with the timestamp, location and the duration spent on the floor prior to standing up. Location might indicate specific risks which has led to a fall, such as a step. Duration spent on the floor provides information on the severity of the fall.

These parameters are transmitted in real-time to a server, where they are processed using deep learning and machine learning algorithms, and then synthesised into a dashboard that is accessible to healthcare professionals responsible for conducting falls risk screening. Additionally, the monitored individual and their family members can have access to the dashboard for continuous self-screening. Upon user request, the system can provide more detailed information. The description and visualisation of parameters are customisable by the user.

2. Detailed Definitions of the Key Concepts

Risk of falls. This concept is the central variable of the falls risk assessment and prevention system map. It reflects the hazard linked to an individual's susceptibility to falling. The overall system map is constructed to explain whether falls risk is influenced and/or caused by other factors and to examine variations in falls risk across diverse scenarios.

Severity of previous falls. The level of severity of previous falls. A fall is severe if it has one of the five characteristics: (1) accompanying injury, (2) multiple falls in the last year, (3) known frailty, (4) inability

to get up after the fall without help for at least an hour, and (5) accompanied by (suspected) transient loss of consciousness. Older adults who have fallen in the last 12 months had a greater history of falls than non-fallers (71.9% vs. 11.9%, p < 0.001[4]). In case of a fall in the last year, the British Geriatrics Society suggests a fall severity assessment[1]. The guidelines designate an individual as 'High risk' in the event of a severe fall, and 'Intermediate risk', otherwise.

Fear of falling. Evaluations of concern about falling are assessed using standardised measures such as the Falls Efficacy Scale International (FES-I[5]) or Short FES-I[6] in community-dwelling older adults. A history of prior falls is associated with a twofold likelihood of experiencing fear of falling compared to individuals without such incidents[7].

Adverse health conditions. This concept represents all the risk factors of falls related to a person's health and wellbeing, other than mobility issues. These factors are summarised in a table for multifactorial falls risk assessment in the World Falls Guidelines[1]. They include problems with sensory functions (dizziness/ vestibular, vision, hearing), cognitive functions (cognition, delirium, behaviour), autonomic functions (orthostatic, hypotension), medications/polypharmacy, nutritional status (malnutrition, obesity, vitamin deficiencies, substance abuse, alcohol use), and disease history such as cardiovascular disorders, contributing diseases (diabetes mellitus, osteoarthritis, neurological disorders), atypical disease presentations of acute conditions (e.g., pneumonia), Parkinson's disease, and depression.

Activities of daily living (ADLs). ADLs are the fundamental skills required to independently care for oneself [8], or one's functional ability[1]. ADLs comprise two types of activities: basic and instrumental. Basic ADLs are related to the basic physical needs such as ambulating, feeding, dressing, personal hygiene, continence, and toileting. Instrumental ADLs require more technical skills such as transportation, shopping, managing finances, meal preparation, home maintenance, house cleaning, communication by telephone and mail, and managing medications[8]. Dependence in basic and instrumental activities of daily living is reported as a predictor of falls in the following year[9]. This variable is defined as the extent to which a person can successfully perform these activities.

Mobility limitations. This concept represents the problems related to mobility such as balance disorders, gait impairment, muscle weakness, frailty, the need for walking aid and potential foot problems (excluding footwear, which is an environmental risk[1]). Mobility limitations and balance disorders were found associated with future falling events in community-dwelling older adults[1,4,10,11].

Environmental risk. This concept encompasses factors contributing to falls in relation to the indoor home environment and footwear. Environmental risks are assessed using tools including the Westmead Home Safety Assessment[12] and the Falls Behavioural Scale for the Older Person[13]. The CDC suggests referring to an occupational therapist to evaluate home safety as well, in their STEADI algorithm to prevent falls[11]. In home environment, insufficient lighting in hallways, stairways, and bathrooms can compromise vision and depth perception, particularly during the night. Uneven surfaces, such as slippery polished floors, loose mats, and throw rugs without non-slip backing, might pose tripping hazards. Studies indicate that frequent fall injuries occur during transitions between carpeted and non-carpeted areas, on wet carpets or rugs, and while rushing to the bathroom[14]. Targeted home modifications have been associated with a reduction in falls and an improvement in activities of daily living[15]. Inappropriate footwear, including bare footedness, has been identified as a risk factor for falls[1]. One suggested intervention is the provision of education focusing on shoe fit, traction, insoles, and heel height[11].

Activity Level. This concept represents the degree to which an individual engages in physical activity as part of their daily routine at home. It is a summarised metric calculated over a specific period, such as a day or a month. When an individual is detected in a room, their status is categorised as either active or inactive. Active states include activities ranging from walking to intensely exercise, while inactive

states refer to periods of being bed-bound or sedentary. During active periods, various parameters such as gait speed, step count, or activity intensity are measured for each minute. The activity level for a day or month is then calculated based on these parameters, providing a comprehensive overview of the individual's physical engagement. Greater levels of physical activity among the older adults correspond to a diminished likelihood of experiencing a fall[16,17].

Falls risk screening. This variable represents the number of falls risk screening procedures and assessments of a person, conducted by a healthcare professional, using falls risk screening tools and methods. There is a wide range of tools and methods with varying sensitivity and specificity. The use of the TUG test and measuring gait speed are recommended by the guidelines[1,2].

Fall reporting accuracy. A fall diary is a way of recording fall frequency and other related information such as its timing, location, symptoms, and injuries[18]. Healthcare providers ask their patients to record their falls to personalise the interventions. This variable is defined as an individual's accuracy in reporting falls. The value of this variable decreases in case of underreporting[19,20].

Opportunistic finding. This variable refers to the strategy of identifying individuals who are at risk of falling when they visit healthcare settings for reasons other than falls. This approach is recommended for community-dwelling older adults and involves healthcare professionals taking the opportunity during any routine healthcare encounter to ask about the number falls in the last year, unsteadiness, or fear of falling[1,21]. This variable indicates the number of observations of a "fall in the last year" by chance, during another interaction with a healthcare provider.

Effectiveness of intervention. This concept is defined as the degree to which implemented interventions successfully reduces the incidence, severity, and consequences of falls, promoting overall well-being and preserving functional independence among older adults. This concept includes the individual's behaviour towards the intervention.

Primary costs to National Health Service (NHS). This concept encapsulates all the primary costs incurred because of a fall such as the financial expenditures incurred for medical services, including hospital stays, physician fees, medications, and treatments, associated with addressing fall-related incidents among individuals within the healthcare system. In the UK, over 3 million people have osteoporosis with a high risk of fragility fractures. Hip fractures alone contribute to 1.8 million hospital bed days and £1.1 billion in hospital expenses annually, excluding the substantial costs associated with social care[22]. The cost of falls is estimated as 3.2 billion per year[23].

Healthcare professionals' workload. This concept reflects the extent of responsibilities, tasks, and time commitments required of medical practitioners, including doctors and nurses, in managing and addressing the healthcare needs, assessments, and interventions related to falls and associated consequences. High workload and work stress are recently reported as the primary factors driving healthcare providers out of the NHS[24].

Societal costs. This variable encompasses the broader economic and social impacts resulting from falls among older adults, including factors such as social care and individuals reducing work hours to provide caregiving support. The total annual social care cost of fragility fractures to the UK is estimated as £1.1 billion[22].

Radar-based continuous home monitoring. This variable represents the proposed system to improve the falls risk assessment and prevention. It is defined as the application of millimetre-wave radar technology within home environments to measure key parameters systematically and continuously such as gait, mobility, activities, and behavioural patterns among older adults, enabling timely and comprehensive insights for falls risk assessment and prevention. Since it is designed to provide continuous falls risk screening and accurate reporting of fall detection, it is proposed to replace "Falls risk screening", "Fall reporting accuracy" and "Opportunistic finding" variables in the system.

3. Adjacency Matrices

Adjacency matrix of the current system map

	Risk of falls	Severity of previous falls	Fear of falling	Adverse health conditions	Activities of daily living	Mobility limitations	Environmental risk	Activity level	Falls risk screening	Fall reporting accuracy	Opportunistic finding	Effectiveness of intervention	Primary costs to NHS	Health care professionals workload	Societal costs
Risk of falls			0.15										0.74	0.78	0.71
Severity of previous falls	0.63		0.67	0.16	-0.34	0.45		-0.24	0.08		0.2		0.22	0.08	0.22
Fear of falling	0.28				-0.14	0.1	-0.2	-0.34		0.12	0.12		0.04	0.14	0.28
Adverse health conditions	0.46		0.18		-0.42	0.12	0.06	-0.2	0.08		0.3	-0.06	0.44	0.22	0.38
Activities of daily living	-0.32		-0.08				-0.08						-0.24	-0.28	-0.54
Mobility limitations	0.64		0.34		-0.36		0.28	-0.34	0.32		0.18	-0.06	0.24	0.34	0.55
Environmental risk	0.41		0.12						0.18				0.06	0.16	0.06
Activity level	-0.4		-0.15		0.28	0.1							-0.16	-0.1	-0.1
Falls risk screening	-0.02		0.04									0.58	-0.02	0.36	0.02
Fall reporting accuracy									0.08			0.56		0.1	0.1
Opportunistic finding									0.31			0.27	0.02		
Effectiveness of intervention	-0.66		-0.34	-0.08	0.38	-0.22	-0.5	0.46					-0.1		
Primary costs to NHS														0.16	
Healthcare professionals workload													-0.02		
Societal costs															

Adjacency matrix of the proposed system map

	Risk of falls	Severity of previous falls	Fear of falling	Adverse health conditions	Activities of daily living	Mobility limitations	Environmental risk	Activity level	Effectiveness of intervention	Primary costs to NHS	Health care professionals workload	Societal costs	Real-time home monitoring
Risk of falls			0.15							0.74	0.78	0.71	
Severity of previous falls	0.63		0.67	0.16	-0.34	0.45		-0.24		0.22	0.08	0.22	
Fear of falling	0.28				-0.14	0.1	-0.2	-0.34		0.04	0.14	0.28	
Adverse health conditions	0.46		0.18		-0.42	0.12	0.06	-0.2	-0.06	0.44	0.22	0.38	
Activities of daily living	-0.32		-0.08				-0.08			-0.24	-0.28	-0.54	
Mobility limitations	0.64		0.34		-0.36		0.28	-0.34	-0.06	0.24	0.34	0.55	
Environmental risk	0.41		0.12							0.06	0.16	0.06	
Activity level	-0.4		-0.15		0.28	0.1				-0.16	-0.1	-0.1	
Effectiveness of intervention	-0.66		-0.34	-0.08	0.38	-0.22	-0.5	0.46		-0.1			
Primary costs to NHS											0.16		
Healthcare professionals workload										-0.02			
Societal costs													
Real-time home monitoring	-0.06		-0.34				-0.14	0.4	0.8	-0.28	-0.43	-0.38	

References

1. Montero-Odasso, M. *et al.* World guidelines for falls prevention and management for older adults: a global initiative. *Age and Ageing* **51**, afac205 (2022).
2. Eckstrom, E. *et al.* American Geriatrics Society response to the World Falls Guidelines. *Journal of the American Geriatrics Society* (2024).
3. Taylor, K. I., Staunton, H., Lipsmeier, F., Nobbs, D. & Lindemann, M. Outcome measures based on digital health technology sensor data: data- and patient-centric approaches. *npj Digit. Med.* **3**, 1–8 (2020).
4. Lee, Y.-Y., Chen, C.-L., Lee, I.-C., Lee, I.-C. & Chen, N.-C. History of Falls, Dementia, Lower Education Levels, Mobility Limitations, and Aging Are Risk Factors for Falls among the Community-Dwelling Elderly: A Cohort Study. *Int J Environ Res Public Health* **18**, 9356 (2021).
5. Yardley, L. *et al.* Development and initial validation of the Falls Efficacy Scale-International (FES-I). *Age and ageing* **34**, 614–619 (2005).
6. Kempen, G. I. *et al.* The Short FES-I: a shortened version of the falls efficacy scale-international to assess fear of falling. *Age and ageing* **37**, 45–50 (2008).
7. Lavedán, A. *et al.* Fear of falling in community-dwelling older adults: A cause of falls, a consequence, or both? *PLOS ONE* **13**, e0194967 (2018).
8. Edemekong, P. F., Bomgaars, D. L., Sukumaran, S. & Schoo, C. Activities of Daily Living. in *StatPearls* (StatPearls Publishing, Treasure Island (FL), 2023).
9. Sasidharan, D. K. *et al.* Incidence and risk factors for falls among community-dwelling elderly subjects on a 1-year follow-up: a prospective cohort study from Ernakulam, Kerala, India. *BMJ Open* **10**, e033691 (2020).
10. Li, Y. *et al.* Risk factors for falls among community-dwelling older adults: A systematic review and meta-analysis. *Frontiers in Medicine* **9**, (2023).
11. Stevens, J. A. The STEADI Tool Kit: A Fall Prevention Resource for Health Care Providers. *IHS Prim Care Provid* **39**, 162–166 (2013).
12. Clemson, L. Home fall hazards and the Westmead home safety assessment. *West Brunswick, Australia: Co-ordinates Publications* (1997).
13. Clemson, L., Cumming, R. G. & Heard, R. The falls behavioural (FaB) scale for the older person. *Sydney: The University of Sydney* (2003).
14. Rosen, T., Mack, K. A. & Noonan, R. K. Slipping and tripping: fall injuries in adults associated with rugs and carpets. *J Inj Violence Res* **5**, 61–69 (2013).
15. Stark, S., Stark, S., Keglovits, M. & Somerville, E. A Randomized Controlled Feasibility Trial of Tailored Home Modifications To Improve Activities of Daily Living. *The American Journal of Occupational Therapy* **70**, 7011520290p1 (2016).
16. Osundiya, O., Adeagbo, C., Giwa, T., Adepoju, F. & Oyedemi, J. 105 Physical Activity Level and its Association with Risk of Fall and Fear of Fall among the Elderly. *Age and Ageing* **48**, iv18–iv27 (2019).
17. Pettee Gabriel, K. *et al.* Physical activity trajectories and subsequent fall risk: ARIC Study. *Preventive Medicine* **121**, 40–46 (2019).
18. Ashburn, A., Stack, E., Ballinger, C., Fazakarley, L. & Fitton, C. The circumstances of falls among people with Parkinson's disease and the use of Falls Diaries to facilitate reporting. *Disability and Rehabilitation* **30**, 1205–1212 (2008).
19. Hoffman, G. J. *et al.* Underreporting of Fall Injuries by Older Adults: Implications for Wellness Visit Fall Risk Screening. *J Am Geriatr Soc* **66**, 1195–1200 (2018).
20. Shorr, R. I. *et al.* Improving the Capture of Fall Events in Hospitals: Combining a Service for Evaluating Inpatient Falls with an Incident Report System. *Journal of the American Geriatrics Society* **56**, 701–704 (2008).
21. Centers for Disease Control and Prevention STEADI. Pocket Guide—Preventing Falls in Older Patients. (2019).
22. Office for Health Improvement and Disparities. Falls: applying All Our Health. *GOV.UK* https://www.gov.uk/government/publications/falls-applying-all-our-health/falls-applying-all-our-health (2022).

23. National Institute for Health and Care Excellence. *Falls in Older People: Assessing Risk and Prevention. Clinical Guideline [CG161]*. https://www.nice.org.uk/guidance/cg161 (2013).
24. Weyman, A. *et al.* Determining the relative salience of recognised push variables on health professional decisions to leave the UK National Health Service (NHS) using the method of paired comparisons. *BMJ Open* **13**, e070016 (2023).
